# Stress sensing and response through biomolecular condensates in plants

**DOI:** 10.1016/j.xplc.2024.101225

**Published:** 2024-12-18

**Authors:** Jiaxuan Peng, Yidan Yu, Xiaofeng Fang

**Affiliations:** 1 Center for Plant Biology, School of Life Sciences, Tsinghua University, Beijing 100084, China

**Keywords:** stress, biomolecular condensates, phase separation, sensor

## Abstract

Plants have developed intricate mechanisms for rapid and efficient stress perception and adaptation in response to environmental stressors. Recent research highlights the emerging role of biomolecular condensates in modulating plant stress perception and response. These condensates function through numerous mechanisms to regulate cellular processes such as transcription, translation, RNA metabolism, and signaling pathways under stress conditions. In this review, we provide an overview of current knowledge on stress-responsive biomolecular condensates in plants, including well-defined condensates such as stress granules, processing bodies, and the nucleolus, as well as more recently discovered plant-specific condensates. By briefly referring to findings from yeast and animal studies, we discuss mechanisms by which plant condensates perceive stress signals and elicit cellular responses. Finally, we provide insights for future investigations on stress-responsive condensates in plants. Understanding how condensates act as stress sensors and regulators will pave the way for potential applications in improving plant resilience through targeted genetic or biotechnological interventions.

## Teaser

Biomolecular condensates are emerging as key players in plant–environment interactions. This review summarizes the functions of plant condensates in stress perception and response and provides insights for future investigations of stress-responsive condensates in plants, highlighting their potential as targets of genetic engineering for improving plant resilience.

## Introduction

Plants encounter various challenges in natural environments. The ability to sense and respond to stress rapidly and effectively throughout their life cycle is crucial for plant adaptation and survival. In turn, plants have developed numerous sophisticated and exquisite mechanisms of stress response, and these mechanisms have been studied extensively in past decades (Jones and Dangl, 2006; Fu and Dong, 2013; Zhu, 2016; van Zelm et al., 2020; Zhou and Zhang, 2020; Yuan et al., 2021; Zhang et al., 2022a). Proteins located on or near the plasma membrane have been identified as key sensors, with ion channels, especially calcium channels, playing a predominant role. Recent studies suggest that biomolecular condensation is a promising mechanism for both stress perception and response.

**Figure 1:**
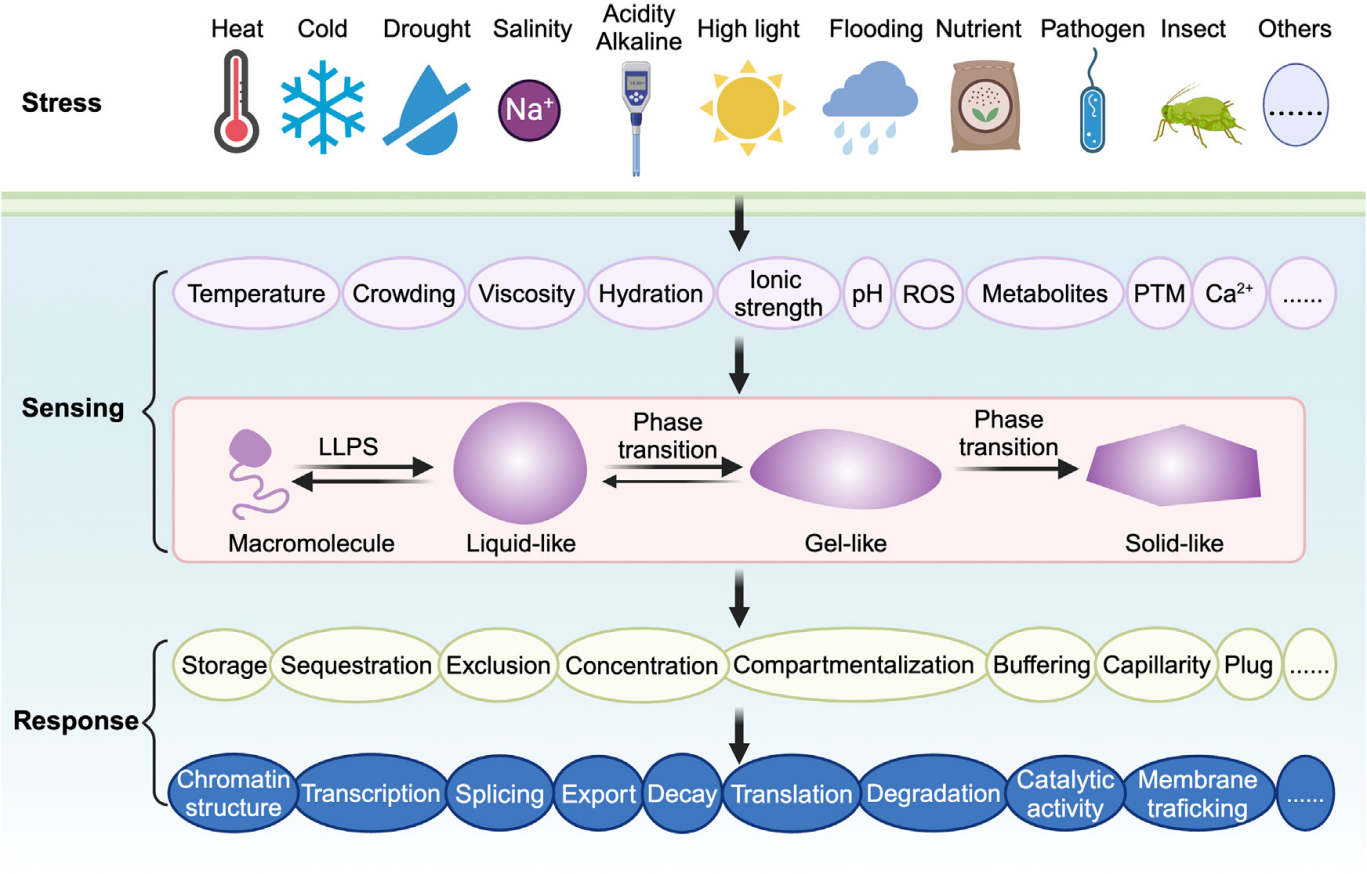
The role of biomolecular condensates in stress perception and response. The schematic illustration shows that various environmental stimuli trigger intracellular changes that can affect the processes of liquid–liquid phase separation and phase transition, which elicit the adjustment of gene expression as well as cellular properties via distinct mechanisms.

Since the initial description of P granules in *C. elegans* in 2009 (Brangwynne et al., 2009), it has been generally recognized that most biomolecular condensates arise from phase separation and/or phase transition (Shin and Brangwynne, 2017). Multivalent interactions between macromolecules are the main driving forces for phase separation. Because most of their amino acids are exposed, intrinsically disordered regions (IDRs) are able to provide weak multivalent interactions through electrostatic interactions, π–π stacking, cation–π interactions, and so forth (Brangwynne et al., 2015). A unique subclass of IDRs known as prion-like domains (PrLDs) have been identified across various organisms and are implicated in mediating phase separation (Franzmann and Alberti, 2019). Lack of a stably folded structure and high solvent accessibility make IDR conformational biases inherently sensitive to physicochemical changes. Therefore, the assembly and disassembly of biomolecular condensates are often susceptible to changes in aspects of the cellular environment such as temperature, pH, redox state, molecular crowding, and ionic strength (Figure 1). Indeed, recent studies have begun to highlight biomolecular condensates as sensors of stress. Biomolecular condensation can modulate cellular activity via distinct mechanisms. These include but are not limited to enhancing or inhibiting biochemical reactions, forming new protein–protein or protein–nucleic acid interaction networks, acting as buffers, and generating capillary forces (Alberti et al., 2019). Thus, biomolecular condensates can also transduce stress signals to downstream processes (Figure 1).

**Figure 2:**
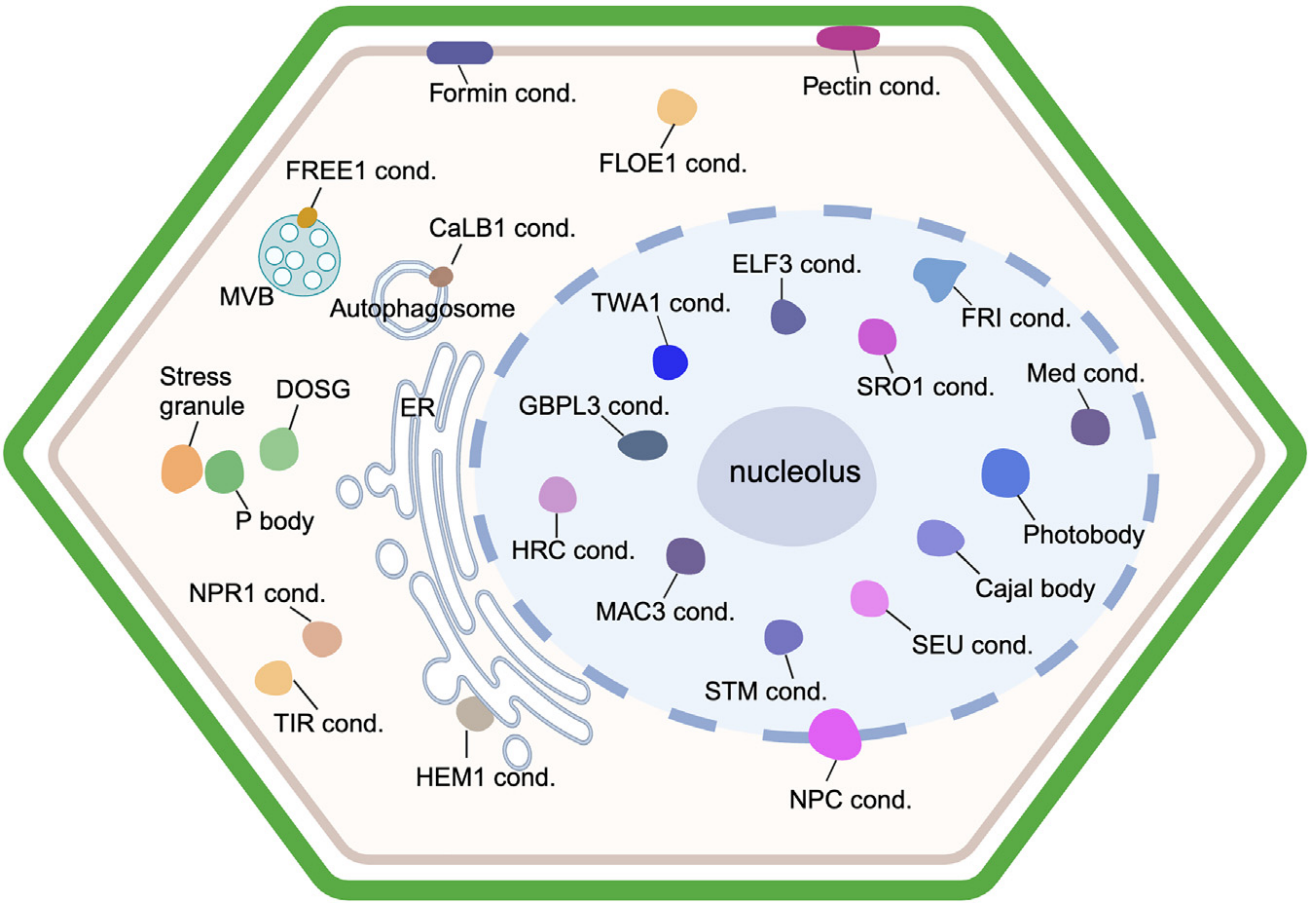
Stress-related biomolecular condensates in plant cells. Schematic of the numerous condensates that participate in perception and response to various stresses in plant cells. Cond., condensate.

A large number of condensates are directly or indirectly involved in stress perception and response in plant cells. These include deeply conserved and well-defined condensates such as stress granules (SGs), processing bodies (PBs), and the nucleolus, as well as more recently documented condensates (Figure 2). In this review, we summarize current knowledge on the role of biomolecular condensates in plant stress perception and response and discuss current questions and future directions in the field. We focus on the condensates formed by plant proteins; those formed by virus or bacterial proteins in plant cells are not discussed.

## Stress granules (SGs)

SGs are a prominent type of conserved cytoplasmic biomolecular condensate that form in response to various stressors (Protter and Parker, 2016; Ivanov et al., 2019). The formation and/or dynamics of SGs are tightly linked to cell survival and pathological phenomena in human diseases. The persistent presence of SGs and/or the imbalance between SG formation and disassembly causes various diseases, such as cancer, cardiovascular diseases, and neurodegenerative disorders (Arimoto et al., 2008; Eisinger-Mathason et al., 2008; Ramaswami et al., 2013; Cui et al., 2023).

### Assembly and disassembly of SGs

In the 1980s, researchers discovered granules in tomato cells after heat treatment, which they termed heat shock granules. These granules predominantly consisted of heat shock proteins (HSPs) and RNAs and were regarded as the earliest evidence of SGs (Nover et al., 1989). Subsequent decades of study defined SGs as transient assemblies of stalled preinitiation complexes, small ribosomal subunits, translationally arrested mRNAs, and various RNA-binding proteins (RBPs) (Ivanov et al., 2019).

In mammalian cells, the formation of SGs is initiated by translational inhibition. Stress induces phosphorylation of the translation initiation factor eukaryotic initiation factor 2 subunit alpha (eIF2α), leading to dissociation of the translation initiation complex from polysomes (Clemens, 1996). This dissociation causes mRNAs to accumulate in ribonucleoprotein (RNP) complexes, facilitating the assembly of SGs (Marcelo et al., 2021). Super-resolution microscopy has revealed that the interior of SGs is not uniform; instead, regions with high concentrations of proteins or RNA are present. Transmission electron microscopy has revealed dense fibrillar patches within the SG ultrastructure, referred to as cores, which are more dense and solid. The surrounding, less-concentrated regions are known as the shell and exhibit greater dynamic properties (Souquere et al., 2009; Jain et al., 2016). However, the mechanism underlying core and shell formation has remained ill-defined.

Evidence from mammalian cells, yeast, and plants has demonstrated the presence of stable interaction networks among core SG proteins, even under non-stress conditions. These pre-existing networks facilitate the rapid formation of SGs upon stress exposure. In non-stressed mammalian cells, SG proteins are also enriched in the RAS GTPase-activating protein-binding protein1 (G3BP1) interactome (Markmiller et al., 2018). In plants, research on the Tudor staphylococcal nuclease (TSN) revealed that approximately 70% of the proteins involved in SG protein–protein interaction networks are already engaged under non-stress conditions (Gutierrez-Beltran et al., 2021; Maruri-López et al., 2021).

It is accepted that SGs assemble via phase separation driven by dynamic and promiscuous protein–protein and protein–RNA interactions (Protter and Parker, 2016). These interactions are distributed unevenly, with the G3BP1/2 nodes more important than others (Yang et al., 2020). A current model holds that G3BP functions as a molecular switch: stalled translation leads to an increase in cellular free RNA concentrations, which, above a certain threshold, triggers a conformational change in G3BP that favors G3BP–RNA interaction. This results in RNA–dependent phase separation that seeds SG assembly (Deniz, 2020; Guillén-Boixet et al., 2020; Sanders et al., 2020; Yang et al., 2020). Interestingly, in U2OS cells that lack G3BP1 and G3BP2, SG formation is reduced under oxidative stress but not under osmotic or heat stress (Kedersha et al., 2016; Yang et al., 2020), suggesting that the mechanism of SG assembly varies under different stresses.

In *Arabidopsis*, general control nonderepressible 2 (GCN2) is the kinase that phosphorylates eIF2α. GCN2-mediated phosphorylation of eIF2α is triggered in response to amino acid starvation, UV exposure, cold stress, injury, and the presence of plant defense hormones such as jasmonic acid and salicylic acid (SA) but not by heat (Lageix et al., 2008). Unlike phosphorylated eIF2α in mammalian cells, which blocks the exchange of guanosine diphosphate (GDP) for guanosine triphosphate (GTP), thereby leading to global translation inhibition (Wek, 2018), studies from wheat germ extract showed that GDP–GTP exchange by eIF2 can be spontaneous and occur independently of phosphorylation state (Shaikhin et al., 1992). As such, phosphorylation of wheat eIF2α only slightly reduces the translation of specific mRNAs (Zhigailov et al., 2020). There is therefore no definitive evidence that supports the necessity of GCN2 and phosphorylated eIF2α for SG formation in plants (Lokdarshi and von Arnim, 2022). The mechanism underlying the initiation of SG assembly in plants remains a mystery.

The composition of plant SGs is largely homologous to that in mammals or yeast (Kearly et al., 2024). RBP47B is the homolog of animal T cell intracellular antigen-1. A recent study showed that RBP47B functions as a sensor for phenolic acids (PAs) secreted by neighboring plants during plant allelopathy. These PAs directly bind to RBP47B, inducing its phase separation and promoting SG formation, thus leading to global translation inhibition (Xie et al., 2023). This provides a hint as to how SGs are assembled in response to biotic stimuli and suggests that the SG assembly mechanism could differ under different stress conditions.

The dynamics of SGs in plants have been characterized primarily under heat-stress conditions. Under mild heat stress (34°C), SGs exhibit high molecular mobility, characterized by rapid and long-distance movements, with a tendency to fuse into larger foci over time, as well as rapid disassembly upon stress recovery. By contrast, SGs formed under higher temperatures (>40°C) are smaller and less mobile (Hamada et al., 2018). A recent study showed that an increase in the temperature or duration of heat stress reduced the mobility of SG components and inhibited SG clearance during stress recovery, likely owing to partitioning of proteasome components inside the SGs (Xie et al., 2024).

### SGs in stress responses

Because SGs are formed via an interaction network, elimination of a single component is usually insufficient to completely abolish their formation. In addition, it remains challenging to discriminate the function of a protein per se from its role in SGs. For example, the major SG scaffold G3BPs can function independently of SGs in regulating mammalian target of rapamycin signaling (Prentzell et al., 2021). These caveats make it difficult to interrogate SG function. Nevertheless, numerous studies have implicated SGs in translational control, biomolecule storage, mRNA sorting and regulation, cell signaling, and inhibition of viral replication (Marcelo et al., 2021).

SG components selectively bind specific transcripts, regulating their stability or translation. Upon stress relief, SG disassembly releases mRNAs and restores translation. This mechanism likely reduces the energy cost of translating non-essential gene products, as excess mRNAs may compete for limited translation factors, thereby lowering the translation efficiency of mRNAs that are essential for survival (Decker and Parker, 2012). In yeast, the RNA helicase DED1 protein (Ded1p) helps ribosomes to scan the 5′ UTR of housekeeping mRNAs. During heat stress, Ded1p condenses into SGs, inactivating itself and repressing the translation of housekeeping mRNAs (Iserman et al., 2020). However, stress-related mRNAs escape translational repression. This is likely because the 5′ UTRs of stress-related mRNAs are short and structurally simple, allowing them to be directly resolved by eIF4A, even when other translation initiation elements are sequestered in SGs (Desroches Altamirano et al., 2024).

SGs have been shown to protect specific mRNAs in plants, including stress-related and housekeeping mRNAs. For instance, the SG component oligouridylate binding protein 1C associates with non-uracil-rich mRNAs during hypoxia, sequestering poorly translated mRNAs into SGs during transient low-energy stress. Upon recovery, the rapid disassembly of SGs enables stabilized mRNAs to reenter translation (Sorenson and Bailey-Serres, 2014). The small dimeric DNA/RNA-binding proteins acetylation lowers binding affinity 4/5/6 (ALBA4/5/6) localize to SGs during heat stress, stabilizing heat shock factor (HSF) mRNAs and enhancing plant thermotolerance in *Arabidopsis* (Tong et al., 2022). It is worth noting that ALBAs are also localized to PBs under heat stress. The double-stranded RNA binding protein drought resistance gene 9 in rice recruits mRNAs of *9-cis-epoxycarotenoid dioxygenase4* (*NCED4*), a key gene for abscisic acid biosynthesis, into SGs and protects them from degradation. Maintenance of *NCED4* expression under drought conditions promotes ABA synthesis, thereby enhancing drought tolerance in rice (Wang et al., 2024a). In addition, TSN1-related SGs bind to mRNA of *gibberellin 20-oxidase 3* (*GA20ox3*), which encodes a key enzyme in gibberellin biosynthesis, thereby increasing *GA20ox3* mRNA levels and improving salt tolerance (Yan et al., 2014). Plant SGs also recruit housekeeping mRNAs. For example, mRNAs encoding ribosomal proteins are preferentially recruited into SGs under heat stress and released in an HSP101-dependent manner during recovery (Merret et al., 2017). It seems that various mRNAs can be recruited into SGs by specific RBPs. Recent studies on bacterial SGs suggest that mRNA stabilization by SGs is achieved by the exclusion of mRNA ribonucleases from SGs, likely owing to charge repulsion between negatively charged mRNAs within SGs and negatively charged ribonucleases (Pei et al., 2024). However, the mechanisms that underlie the stabilization of mRNAs by eukaryotic SGs remain unclear. Gathering more information on the complete composition of SGs and reconstitution of SGs *in vitro* should help us to understand this.

Partitioning of mRNAs by plant SGs also affects translation. A recent study reported that the condensation of glycine-rich RBP 7 (GRP7) into SGs at warm temperature (15–30 min at 38°C) recruited a few translation factors and mRNA chaperones, consistent with the reduced translation of a subset of mRNAs. GRP7 condensates disassembled upon transfer to a lower temperature, releasing the imposed translational inhibition (Xu et al., 2024). The formation of SGs was also correlated with SA-induced translational shutdown (Xie et al., 2023). Nevertheless, there is far from sufficient evidence to support the role of SGs in translational inhibition. The interior of SGs is not by default translationally inactive. In fact, active translation of specific mRNAs has been documented in mammalian cells (Mateju et al., 2020). Whether and to what extent SGs regulate translational arrest and the causal relationship between them warrant further investigation.

Profiling of the SG transcriptome revealed that roughly 10% of bulk mRNA molecules accumulate in mammalian SGs (Khong et al., 2017). In *Arabidopsis*, two RBPs, RNA-binding glycine-rich D2 and D4, are recruited to SGs through phase separation under heat stress, promoting their association with additional SG components and heat-responsive transcripts (Zhu et al., 2022). However, the fate of these heat-responsive transcripts remains to be determined. In addition to the aforementioned cases, the types and fates of most SG-associated RNAs are not well understood; they may be stress specific and vary considerably between studies.

SGs may regulate signaling and metabolism by partitioning key enzymes. Using biochemical fractionation and purification, researchers characterized the SG proteome in *Arabidopsis* seedlings under heat stress. Thirty-three of the identified proteins were conserved across mammals and yeast, whereas the remaining 85 were stress-related proteins, including oxidative stress enzymes, mitogen-activated protein kinase kinase 5 (MKK5) and MPK3, abiotic stress-induced SNF-related kinase (SnRK), and enzymes involved in ethylene, glucosinolate, azelaic acid, and rhamnose metabolism. Interestingly, cyclin-dependent kinase A;1 (CDKA;1) also localized to SGs during stress (Kosmacz et al., 2019). Whether SG localization modulates the kinase activity of CDKA;1 remains to be determined. This mechanism may be conserved, as target of rapamycin complex 1 (TORC1), a kinase involved in yeast cell growth and metabolism, is also sequestered into SGs under heat stress, thus downregulating TORC1 signaling to reduce heat-induced DNA mutations (Takahara and Maeda, 2012). SGs also sequester the rate-limiting enzyme of lariat intronic RNA decay, RNA debranching enzyme 1, under heat stress, resulting in overaccumulation of lariat intronic RNAs in *Arabidopsis* (Wu et al., 2024). SG formation can modulate signaling pathways by altering protein–protein interactions under stress. The SG component TSN appears to mediate SnRK1α condensation into SGs, promoting its heat-induced activation (Gutierrez-Beltran et al., 2021). The DEAD-box RNA helicase RH31 translocates from the nucleus to SGs under salt stress, influencing the transcript levels of some salt-inducible genes (Liu et al., 2021). How SGs regulate metabolism and how metabolites in turn regulate SG assembly are worth investigating in the future (Miao et al., 2024).

## Processing Bodies (PBs)

PBs are cytoplasmic RNP granules composed primarily of proteins and mRNAs involved in translational inhibition and 5′→3′ mRNA degradation (Luo et al., 2018).

### PB assembly

Key markers of PBs in *Arabidopsis* include components of the decapping complex, including decapping proteins 1, 2, and 5 (DCP1, DCP2 and DCP5), VARICOSE (VCS), LIKE-SM (LSM), the 5′-exoribonuclease exoribonuclease 4 (XRN4), and deadenylases such as poly(A)-specific ribonuclease (PARN), and carbon catabolite repression 4A (CCR4a) (Maldonado-Bonilla, 2014). Recent applications of proximity-based labeling also identified RNA helicases as PB components (Liu et al., 2023a).

Protein–protein interaction networks between PB components are well established for yeast and mammalian cells, although the precise hierarchical relationships that underlie PB assembly are not fully understood. In humans, the arginine-glycine-rich domain of human Lsm4 and dead-box helicase 6 (DDX6) (yeast DExH-box helicase 1, DHH1 homolog) are critical for PB maintenance (Ayache et al., 2015; Arribas-Layton et al., 2016), suggesting that a low-complexity sequence may drive PB assembly via phase separation. A recent study reported that ubiquitination of human HAX1 triggers its phase separation and co-condensation with Lsm14A and DDX6, which is essential for PB assembly during energy stress (Zhan et al., 2024). In yeast, the decapping complex subunits Dcp1, Dcp2, adaptor protein enhancer of mRNA decapping 3, and P-body assembly WD repeat protein co-condense *in vitro* at near physiological concentrations in the presence of RNAs (Schütz et al., 2017), likely representing minimal reconstitution of PB assembly. The yeast ATP-dependent RNA helicase DHH1 undergoes phase separation in an ATP- and RNA-dependent manner, facilitating PB nucleation (Mugler et al., 2016). *Arabidopsis* has three DHH1 homologs, RH6, RH8, and RH12, all of which localize to PBs (Chicois et al., 2018). These RHs contain N-terminal glutamine- and proline-rich regions that are intrinsically disordered, making them good candidates for drivers of phase separation. Indeed, *rh6/8/12* mutant plants have significantly fewer PBs (Chantarachot et al., 2020), suggesting that the DHH1/DDX6-like RNA helicases play a conserved role in PB assembly.

*Arabidopsis* DCP5 may also contribute to PB formation. The *dcp5-1* knockdown mutant displays smaller PBs with irregular shapes and defective mRNA decapping (Xu and Chua, 2009). DCP5 undergoes phase separation both *in vivo* and *in vitro*, with two PrLDs being essential (Wang et al., 2023b). Other proteins that localize to PBs may also participate in PB assembly via phase separation. For example, argonaute 1 (AGO1) exhibits a propensity for phase separation *in vivo* and *in vitro* via N-terminal PrLD. In *Arabidopsis* root tip cells, AGO1 does not co-localize with DCP1 under heat stress or normal conditions. However, it does co-localize with other classical PB components, such as DCP5 and UP-frameshift 1 (Blagojevic et al., 2024). It should be noted that PB components can have functions independent of PBs. One such example is the core component DCP1, which localizes to unique plasma membrane subdomains and contributes to cell polarity independently of its role in decapping (Liu et al., 2023a).

### Functions of PBs during stress

Because PBs are enriched with RNA decay factors, the default function of PBs was thought to be mRNA decay and quality control. A recent study characterized the RNA composition of PBs and found that RNAs involved in developmental processes such as cell wall formation, regeneration, and hormonal signaling were enriched in PBs. Counterintuitively, it appears that only RNAs in small-sized PBs are degraded, whereas those in larger PBs are stored, consistent with conclusions from previous *in vitro* studies (Tibble et al., 2021; Liu et al., 2024a). We should bear in mind that this is an interesting correlation, but more direct biochemical and genetic evidence is needed to dissect the causal relationship between PBs and RNA decay, as well as the underlying mechanisms.

In contrast to SGs, PBs are present under normal growth conditions (Xu et al., 2006; Goeres et al., 2007). Under certain stress conditions, the size and number of PBs increase, and their composition is likely also altered (Gutierrez-Beltran et al., 2015), suggesting that PBs also participate in stress responses. For instance, heat treatment induces condensates labeled by both DCP1 and DCP2. Given that DCP1 and DCP2 form the auxiliary and catalytic core subunits of the decapping machinery, respectively, the formation of heat-induced DCP1–DCP2 condensates suggests that a subset of mRNAs detrimental to heat adaptation may be degraded (Motomura et al., 2015). Approximately 25% of the *Arabidopsis* seedling transcriptome has been reported to undergo rapid degradation during early heat stress, facilitating the swift and global reprogramming of gene expression patterns. The cytoplasmic exoribonuclease XRN4 and its co-factor LA-related protein 1A interact within PBs and are crucial for this heat-induced degradation process and essential for plant survival during prolonged exposure to moderately high temperatures (Merret et al., 2013). However, upon short-term heat shock, *xrn4* mutant plants exhibited better heat tolerance, lower leaf surface temperatures, and increased transpiration rates, suggesting that PBs might have distinct roles under different heat-stress conditions (Nguyen et al., 2015). In addition, because XRN4 is an ethylene regulator, *xrn4* exhibits an ethylene-insensitive phenotype (Potuschak et al., 2006), potentially making it more resistant to prolonged heat stress.

The absence of LSM proteins, which are conserved PB components, leads to impaired salt tolerance. This regulation depends primarily on the interactions between LSMs and specific stress-induced transcripts that are targeted for de-capping and subsequent degradation (Zhang et al., 2011; Cui et al., 2014). The DHH1/DDX6-like RNA helicases facilitate mRNA decapping. The DHH1/DDX6-like RNA helicase RH12 was reported to localize in PBs, facilitating the decay of specific germination-related transcripts, and is necessary for salt tolerance during seed germination (Yuan et al., 2024). The *Arabidopsis* BEACH domain-containing protein SPIRRIG (SPI) interacts with DCP1 and localizes to PBs during salt stress. In the salt-sensitive *spi* mutant, salt-stress-related assembly of PBs is impaired (Steffens et al., 2015). Under osmotic stress, rapidly accelerated fibrosarcoma (Raf)-like kinases localize and physically interact with subclass I SnRK2s in PBs, in which the SnRK2s are activated by the Raf-like kinases (Soma et al., 2020). Because the PB component VCS is a direct target of SnRK2 kinases, PBs likely represent a quality control site for the osmotic stress transcriptome. Indeed, many stress-responsive genes were misregulated in *VCS*-knockdown plants (Soma et al., 2017).

Although several lines of evidence support the role of PBs in stress response, it remains to be determined to what extent PBs control the transcriptome under stress and how the specificity is defined. These questions will benefit from future biochemical reconstitution.

### Cross-talk between SGs and PBs under stress

High-resolution microscopy has revealed that some SGs form around PBs after heat treatment, and fusion between SGs and PBs has also been observed (Hamada et al., 2018). In the yeast SG core proteome, 28 proteins have been found to overlap with PB components. In mammalian cells, proximity interaction analyses of known SG and PB proteins also showed that many proteins are shared between SGs and PBs (Youn et al., 2018, 2019). SGs and PBs may interact and exchange components, as evidenced by co-localization of some tandem zinc finger proteins with both SGs and PBs in plants (Pomeranz et al., 2010; Jan et al., 2013). The aforementioned SG components ALBA and TSN are also localized in PBs (Gutierrez-Beltran et al., 2015; Tong et al., 2022). In addition, small molecules contribute to the dynamic behavior of both SGs and PBs. For example, the RNA degradation product 2′,3′-cyclic adenosine monophosphate, a signaling molecule that interacts with RBP47B, induces SG formation (Kosmacz et al., 2018), modulates the abundance of key SG proteins, and enhances PB dynamics (Chodasiewicz et al., 2022). However, the physiological relevance of cross-talk between SGs and PBs is not yet fully understood.

## Nucleolus

The nucleolus is known as the site for RNP synthesis and early stages of ribosome biogenesis (Scherl et al., 2002). The nucleolus has distinctive biophysical properties, including a higher density compared with the surrounding nucleoplasm, which enabled its direct observation in plant and animal cells under an optical microscope in the late eighteenth century (Schöfer and Weipoltshammer, 2018). It can also be isolated through biochemical methods (Scherl et al., 2002).

### Assembly of the nucleolus

The nucleolus exhibits a tripartite structure that facilitates the sequential production of ribosomes from the inner to the outer regions (Lafontaine et al., 2021). Ribosome DNAs within the fibrillar center (FC) are transcribed by Pol I into a 47S rRNA precursor, which is then processed in the dense fibrillar component (DFC) and granular component (GC) to generate mature 18S, 5.8S, and 28S rRNAs by splicing of the unnecessary spacers. The 5S rRNA, produced by Pol III in the nucleoplasm, is subsequently transported into the nucleolus. These rRNAs then assemble with ribosomal proteins to form the 40S and 60S subunits, which are exported from the nucleolus to the cytoplasm (Hua et al., 2022). Multiple copies of both FCs and DFCs are present within a single GC, and their numbers vary across different cell types. The plant nucleolus contains a significantly larger amount of DFC than the typical animal nucleolus (Brown and Shaw, 2008).

Early studies on the nucleolus revealed phenomena of rapid molecular exchange, such as the fusion of two nucleoli and the rapid recovery rate of the GC phase after photobleaching (Phair and Misteli, 2000; Brangwynne et al., 2011). This evidence supports the idea that nucleolus assembly is driven by phase separation. *In vitro* studies have shown that representative proteins, such as fibrillarin (FIB) from the DFC phase and nucleophosmin (NPM1) from the GC phase, can undergo phase separation, forming distinct yet coexisting phases, consistent with *in vivo* observations, suggesting that the layered structure of the nucleolus results from multiphase liquid immiscibility (Feric et al., 2016). The maintenance of multiple phases depends on differences in surface tension between various liquid phases, and IDRs facilitate the condensation of proteins into droplets (Feric et al., 2016). Simultaneously, RNA-binding domains contribute to compartmental specificity by preventing the merging of droplets from different phases. This spatial separation creates reaction chambers for different steps of ribosome maturation, enhancing efficiency and selectivity (Lafontaine et al., 2021).

The distribution of plant ribosome biogenesis proteins in various compartments is similar to that of yeast, suggesting that the overall assembly mechanism is conserved (Palm et al., 2016). The three main rRNA-related nucleolar proteins involved in ribosome biogenesis are FIB, nucleolin, and B23 (also known as nucleophosmin), which are conserved between animals and plants (Kalinina et al., 2018). In mammalian cells, establishment of the DFC phase depends on the FIB protein. FIB self-associates through its IDR, which is rich in glycine–arginine repeats (Yao et al., 2019). In *Arabidopsis*, there are two *FIB* genes, both of which are functional and localize to the nucleolus. Both FIB1 and FIB2 contain a conserved N-terminal R/G domain (Emenecker et al., 2020). Although there has been limited research on the mechanism of nucleolar assembly in plants, this conserved sequence and function across species suggest that phase separation also underpins nucleolar organization in plants.

### Nucleolar stress response

The nucleolus, lacking a membrane, maintains a dynamic exchange with the outer nuclear space, making it an ideal site for regulating cellular homeostasis. Perturbations in ribosome biogenesis, such as nutrient starvation, hypoxia, heat shock, and chemical inhibition of ribosome formation, trigger a cellular stress response known as the nucleolar stress response (Boulon et al., 2010). This response is typically accompanied by changes in nucleolar size and morphology and leads to cell-cycle arrest, senescence, or apoptosis.

In animals, key components of the nucleolar stress response include tumor protein p53 and mouse double minute 2 homolog (MDM2). The nucleolus acts as a stress sensor that selectively activates p53, triggering the transcription of stress-related genes. Under stress conditions, nuclear components, such as NPM1 from the GC phase and ribosomal proteins like ribosomal protein L5 and 11 (RPL5 and RPL11), rapidly redistribute to the cytoplasm. These proteins directly bind to the ubiquitin ligase MDM2, stabilizing p53 (Kurki et al., 2004; Holmberg Olausson et al., 2012). However, unlike animals, plants lack p53 and MDM2 homologs, suggesting that their nucleolar stress response mechanisms may be different (Huart and Hupp, 2013).

In plants, multiple NAC (NAM/ATAF/CUC) transcription factors, which have important roles in the control of biotic and abiotic stress tolerance (Nuruzzaman et al., 2013), are thought to function similarly to p53 in responding to ribosome stress. One candidate, ANAC082, has been identified as a key player in ribosomal stress response. Loss-of-function mutations in ANAC082 mitigate the phenotypic effects of temperature-sensitive ribosome processing defects and confer mild resistance to chemicals that disrupt ribosome biogenesis or function (Ohbayashi et al., 2017). *ANAC082* mRNA contains a conserved upstream open reading frame (uORF) within its 5′ UTR. Typically, the translation of uORFs takes precedence over major ORFs (mORFs), competing for the translation initiation complex and inhibiting mORF translation. (Niu et al., 2020). During nucleolar stress, impaired ribosomal function inhibits uORF translation, resulting in increased ANAC082 expression, making it a promising factor in nucleolar stress-response pathways (Takahashi et al., 2012).

### The nucleolus is a quality-control compartment during stress

Upon stress caused by conditions such as heat, DNA damage, acidosis, and proteasome inhibition, nuclear and cytoplasmic proteins translocate into the nucleolus (Latonen, 2019). These proteins form intranucleolar structures that are referred to as intranucleolar bodies or aggresomes, but the nature and exact mechanism of these structures are not fully clear. These observations led to the hypothesis that the nucleolus serves as an active regulatory site for the detention of extranucleolar proteins. The shape of the plant nucleolus has been shown to undergo alterations in response to stress. The plant nucleolus shows speckled structures upon incubation at 37°C and starts to disaggregate and dissemble after more prolonged exposure (Hayashi and Matsunaga, 2019; Darriere et al., 2022). By contrast, chilling temperatures lead to the formation of a round structure in the nucleolus (Hayashi and Matsunaga, 2019). Under cold stress in soybean root cells, this is manifested by a loose nucleolar structure and an increase in nucleolar size, together with a reduction in the number of FCs and DFCs and a decrease in FIB and B23 protein levels (Stepiński, 2009). In these processes, in which proteins serve as the main components of the response, their functions remain unclear.

Recently, a study in mammalian cells revealed that the liquid-like GC phase of the nucleolus functions as a non-membrane-bound protein quality control compartment under stress conditions (Frottin et al., 2019). Misfolded proteins entered the GC phase of the nucleolus through transient associations with nucleolar proteins such as NPM1, which conferred low mobility to misfolded proteins, preventing irreversible aggregation and maintaining their competence for Hsp70-assisted refolding (Frottin et al., 2019) or disposal by chaperones and proteasomes (Chen et al., 2021). Approximately 200 proteins were identified as being reversibly partitioned upon stress into the immobile substrate of the GC phase, the function of which awaits further investigation. Prolonged stress led to a transition of the GC phase from a liquid-like to a solid state, with loss of reversibility and nucleolar dysfunction (Frottin et al., 2019).

Whether the nucleolus also serves as a compartment for protein storage and quality control under stress is largely unexplored in plants. Given the unique requirement of plants to interact with and defend against environmental stress, we believe that the role of the plant nucleolus in stress responses is more general and widespread than previously anticipated. Future work should systematically characterize the proteins that relocalize into the nucleolus under particular stresses and investigate the effect of nucleolar localization on their functions.

In addition to the above-mentioned condensates, a number of new stress-related condensates have been discovered in the last 5 years. We categorize them below according to the nature of the stress.

## Condensates in temperature sensing and response

The perception of temperature by plant cells is widely distributed throughout subcellular compartments and transcriptional or translational regulatory nodes (Antoniou-Kourounioti et al., 2018; Jung et al., 2023; Kan et al., 2023; Kerbler and Wigge, 2023). Higher plants contain transmembrane calcium channels, such as cyclic nucleotide-gated channels (CNGCs), analogous to the animal thermosensory channel transient receptor potential vanilloid-1 (Guihur et al., 2022). At normal growth temperatures, CNGC channels are closed. Under heat stress, an increase in the fluidity of the surrounding plasma membrane likely induces the opening of CNGC channels, mediating the entry of periplasmic calcium into the cytoplasm (Saidi et al., 2009; Finka et al., 2012; Gao et al., 2012). Through signaling pathways that remain unclear, the HSFs are phosphorylated and translocated to the nucleus where they activate the transcription of HSP-encoding genes (Guo et al., 2016; Ohama et al., 2016). In rice, thermo-tolerance 3.1 (TT3.1) was proposed as a potential heat-stress sensor (Zhang et al., 2022b). TT3.1 is localized on the plasma membrane and relocalizes to endosomes upon heat stress, where it sorts TT3.2 for vacuolar degradation. The accumulation of a small amount of mature TT3.2 in chloroplasts is critical for the protection of thylakoid membranes from heat stress. TT3.1 thus transduces the heat-stress signal from the plasma membrane into intracellular organelles (Zhang et al., 2022b). However, whether and how TT3.1 directly senses heat is unknown.

Temperature is a physical factor that can pass through the plasma membrane; thus, temperature changes can be distributed inside the cellular space. For this reason, temperature perception may not always occur on the cell surface. Cells can perceive different temperatures at intracellular regions and transduce the resulting signal into widely divergent physiological and developmental responses (Vu et al., 2019). Recently, the reversible condensation of proteins has emerged as a crucial mechanism of thermo-sensing and response in plants.

**Figure 3:**
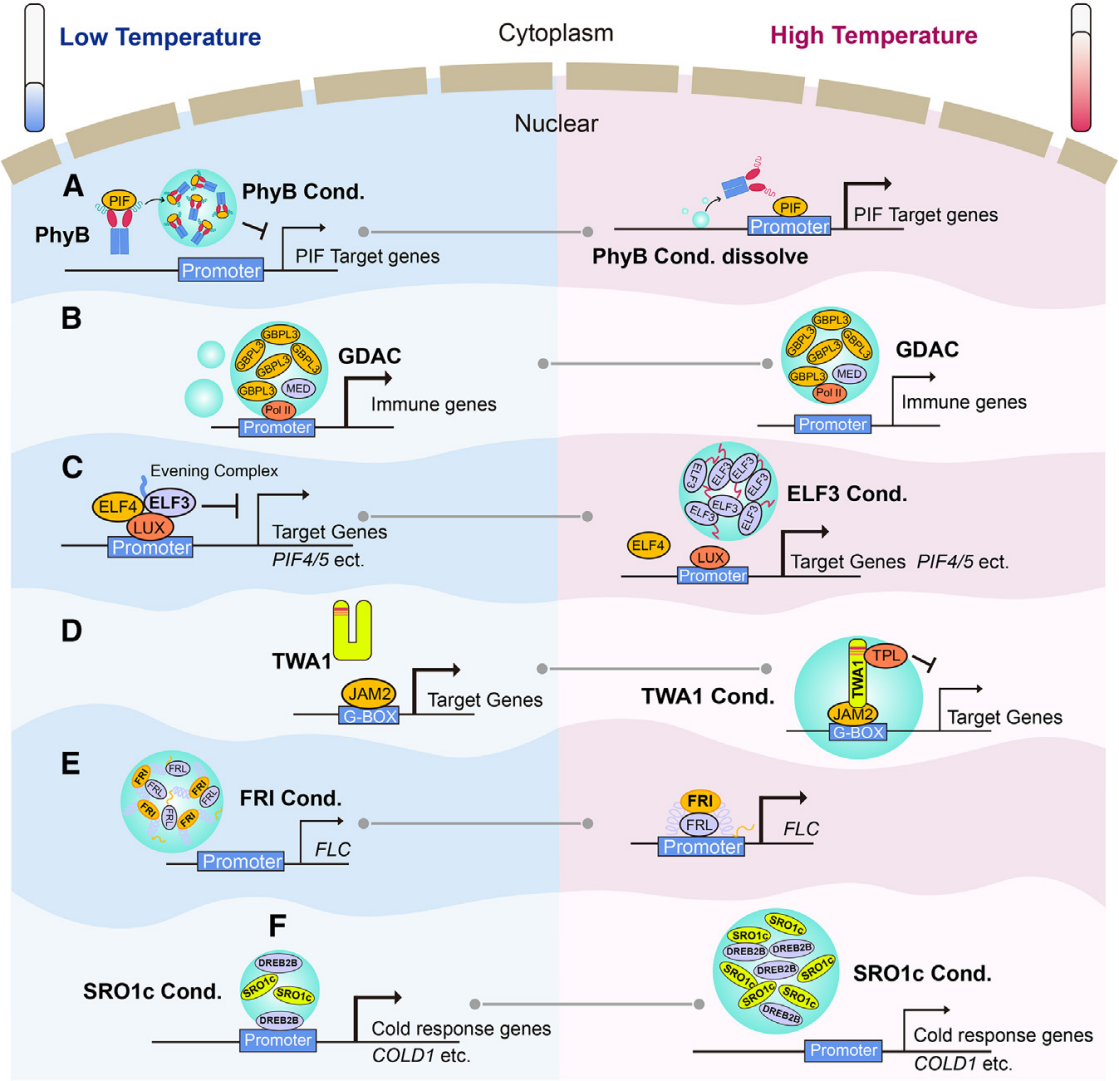
Condensates in temperature sensing and response. **(A)** At low temperatures, phyB condenses and sequesters PIF transcription factors. At high temperatures, phyB condensates disassemble, releasing PIFs and thus promoting PIF-related gene transcription. **(B)** At low temperatures, the GDACs formed by GBPL3 recruit Mediator complex and Pol II around the promoters of immunity-related genes and promote SA production; at high temperatures, the number of GDACs and the association of GBPL3 with gene promoters decrease, leading to impaired plant immunity. **(C)** At low temperatures, the evening complex represses the transcription of target genes; at high temperatures, the evening complex component ELF3 condenses to sequester itself and release the transcriptional repression. **(D)** At high temperatures, TWA1 condenses with jasmonate-associated MYC-like (JAM) transcription factors and the topless (TPL) co-repressors for repressor complex assembly, which suppresses target gene transcription and subsequently upregulates transcription of *HSFA2* and heat shock genes. **(E)** At low temperatures, FRI condenses with FRI-like 1 (FRL1), which is sequestered away from *FLC* loci; at high temperatures, FRI condensates disassemble to upregulate *FLC* transcription. **(F)** At low temperatures, rice SRO1c condenses with DREB2B and upregulates cold-related gene expression by smaller but distinct condensates; at high temperatures, SRO1c condensates become larger but less active for transcription.

Plants make morphological adaptations to warm ambient temperatures, a process termed thermo-morphogenesis (Casal and Balasubramanian, 2019). In *Arabidopsis*, the red/far-red photoreceptor phytochrome B (phyB) links light and temperature perception. In the dark, the active form of phyB (Pfr) converts into its inactive form (Pr), a process accelerated by warm temperatures, thus defining phyB as a thermosensor (Jung et al., 2016; Legris et al., 2016). At lower temperatures, phyB spontaneously condenses into photobodies, a process driven directly by its N-terminal extension and independent of the Pfr-to-Pr dark reversion. These photobodies appear to sequester PIF transcription factors, leading to their degradation and suppressing the transcription of PIF target genes (Chen et al., 2022). At high ambient temperatures, phyB condensates disassemble, releasing this repression and promoting hypocotyl elongation in seedlings (Figure 3A) (Shi and Zhong, 2023). The defense-activated biomolecular condensates (GDACs) formed by an immune regulator, guanylate-binding protein-like GTPase 3 (GBPL3) (detailed in condensates in biotic stress), were also reduced by higher ambient temperature (Figure 3B). Reduced condensation led to the downregulation of *calmodulin binding protein 60G* and *SAR deficient 1*, which encode master immune transcription factors, thereby suppressing SA production and weakening plant immunity (Kim et al., 2022). By contrast, early flowering3 (ELF3), a component of the evening complex, forms condensates at high ambient temperatures (Figure 3C). Because ELF3 represses target gene transcription, its condensation is thought to prevent ELF3 from binding to target-gene promoters, thereby relieving transcriptional repression and promoting flowering. The C-terminal polyglutamine (polyQ) prion domain of ELF3 is responsible for this behavior, with the length of the polyQ repeats correlating with thermal responsiveness (Jung et al., 2020).

When temperatures are above the ambient threshold, plants initiate heat shock responses (Hayes et al., 2021). Recently, a highly disordered nuclear protein, thermo-with ABA-response 1 (TWA1), was found to condense into nuclear subdomains upon heat shock via its N-terminal highly variable region (Figure 3D). Interestingly, natural variation in this region between TWA1 orthologs was correlated with the temperature thresholds required for TWA condensation. TWA1 undergoes a conformational switch that enables its interaction with jasmonate-associated MYC-like (JAM) transcription factors and the topless (TPL) co-repressors for assembly of the repressor complex (Bohn et al., 2024). This complex subsequently controls the transcription of *HSFA2* and other *HSPs*, enhancing thermotolerance.

Many plants require prolonged cold exposure to establish floral competency, a process known as vernalization (Whittaker and Dean, 2017). During vernalization, cold inhibits expression of the central floral repressor gene, *flowering locus C* (*FLC*). In this process, the *FLC* activator frigida (FRI) forms nuclear condensates that rely on its C-terminal IDR and two coiled-coil domains (Figure 3E). These FRI condensates, together with interactors like FRI-like 1, are sequestered away from *FLC* genomic loci, leading to *FLC* repression. These cold-induced FRI condensates are reversibly regulated by warm temperatures, preventing premature flowering during fluctuating autumn temperatures (Zhu et al., 2021). The molecular mechanism by which cold triggers FRI condensation is not yet understood.

The similar to RCD one (SRO) protein family is involved in responses to various abiotic stresses. A recent study found that rice SRO1c plays a key role under cold stress. SRO1c undergoes phase separation and recruits dehydration-responsive element-binding protein 2B (DREB2B) into nuclear condensates (Figure 3F). This SRO1c–DREB2B complex directly responds to low temperatures through dynamic phase transitions, forming smaller nuclear condensates that confer cold tolerance via transcriptional regulation of key cold-tolerance genes, including *chilling-tolerance divergence 1* (Hu et al., 2024).

The role of biomolecular condensates in temperature perception is conserved in other systems. Yeast cells, as unicellular organisms, are exposed to environmental temperature changes. The condensation of proteins, including the polyA-binding protein Pab1 and the translation initiation factors Ded1p and eIF4F, has been reported to play key roles in thermosensing (Riback et al., 2017; Iserman et al., 2020; Desroches Altamirano et al., 2024).

The mechanism of temperature perception is a long-standing question. We are now in an exciting era when this question may be resolved by characterizing the involvement of phase separation. However, we are very early in this path; the atomic basis for temperature perception by phase separation remains unsolved. Temperature is a physical factor; thus, the response to temperature by proteins should lie within the behavior of atoms and follow the theories of physics. Approaches such as computational simulation and biophysical measurements should aid our understanding of this phenomenon.

## Condensates in osmotic and/or salt stress perception and response

Environmental stressors such as drought, cold, and salinity lead to high osmolarity. Hyperosmotic stress elicits changes in cell wall homeostasis (Colin et al., 2023), reductions in cell volume and concomitant changes in membrane mechanics, increased intracellular ionic strength and macromolecular crowding (Ishihara et al., 2021), and loss of hydrating water molecules. Early osmotic signaling in plant cells has been explored intensively over the last decades (Yoshida et al., 2014; Fàbregas et al., 2020). Recently, studies have revealed that the condensation of certain proteins can perceive and respond to changes caused by hyperosmotic stress, therefore playing crucial roles in the osmotic stress signaling pathway.

**Figure 4:**
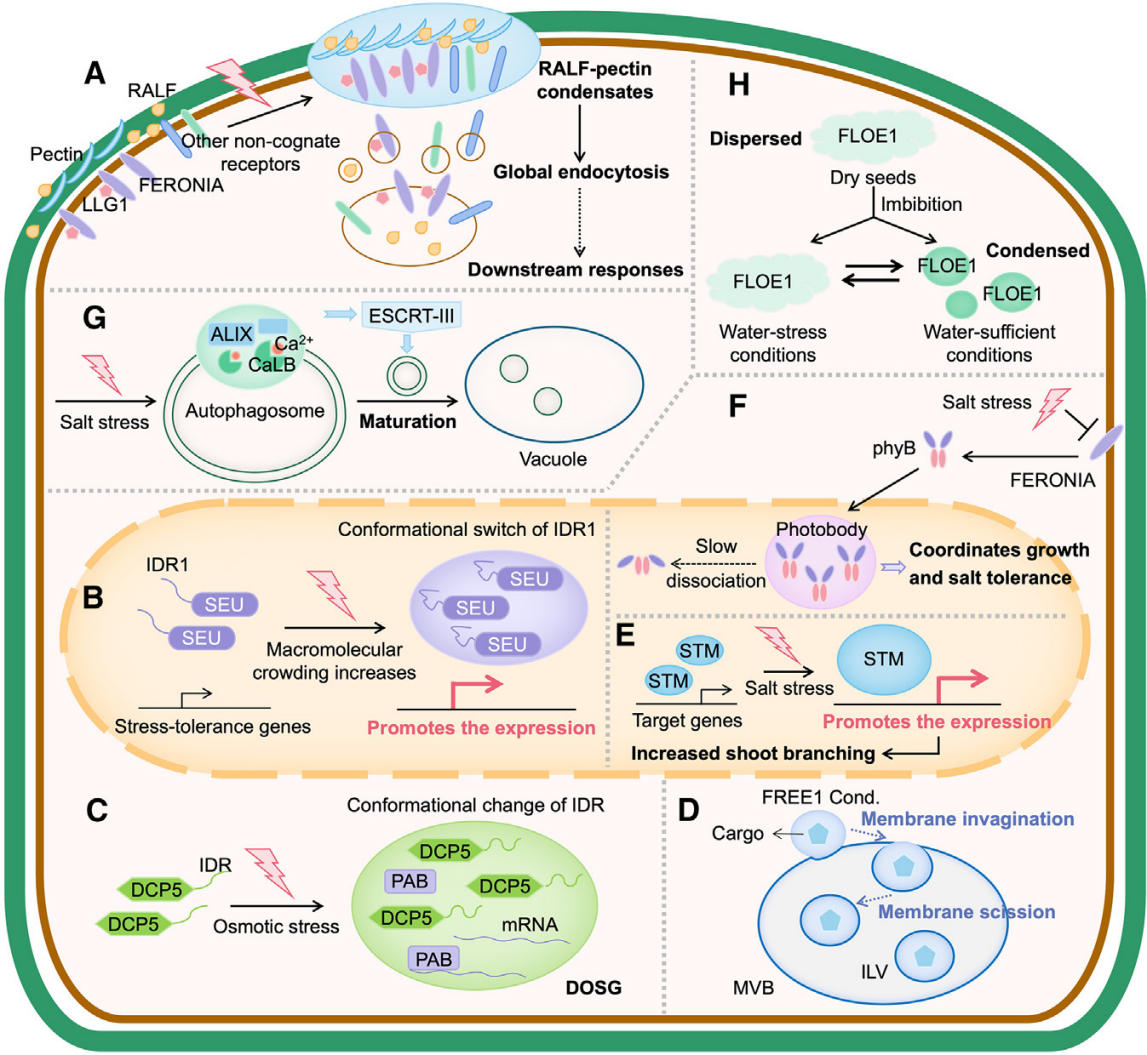
Condensates in water-related stress. **(A)** RALF and pectin undergo extracellular co-phase separation under salt treatment, subsequently recruiting FER–LLG1 and other non-cognate cell surface receptors into the condensates and inducing their endocytosis, which influences downstream signaling pathways and responses to salt stress. **(B)** SEU forms osmotic stress-induced condensates in the nucleus, which depend on the open-to-closed conformational switch of IDR1, subsequently promoting the expression of genes related to the osmotic stress response. **(C)** DCP5 forms molecular crowding-dependent condensates that recruit a subset of mRNAs and regulatory proteins and subsequently modulate the transcriptome and translatome in response to osmotic stress. **(D)** FREE1 condensates can directly invaginate and scission membranes to facilitate ILV formation via surface tension. **(E)** The condensation of STM is enhanced under salt stress, promoting its transcriptional activity and leading to increased shoot branching as an acclimatory response. **(F)** Photobody dissolution is delayed under salt stress by inhibition of FER kinase, thereby fine-tuning the balance between growth and stress tolerance. **(G)** Calcium-dependent lipid binding protein 1 (CaLB1) forms salt-induced condensates on immature autophagosomes with ALG2-interacting protein X (ALIX), which subsequently recruits ESCRT-related proteins and modulates autophagosome maturation. **(H)** FLOE1 forms cytoplasmic condensates upon imbibition under water-sufficient conditions, whereas these condensates become dispersed during imbibition under water-stressed conditions such as salt or osmotic stress.

FERONIA (FER) is a receptor kinase that collaborates with the co-receptor LRE-like GPI-AP1 (LLG1) and the peptide ligand rapid alkalinization factor (RALF) to modulate numerous cellular signaling pathways, helping plants to better grow and survive in dynamic environments (Li et al., 2015; Xiao et al., 2019; Blackburn et al., 2020; Cheung, 2024). A recent study shed light on the regulatory mechanisms by which RALF–FER–LLG1 orchestrate cellular processes (Liu et al., 2024b). The authors found that salt treatment triggers extracellular co-phase separation of RALF with pectin (Figure 4A), a component of the plant cell wall. RALF–pectin condensates then recruit the cognate receptor FER–LLG1, as well as numerous other non-cognate cell surface receptors, into the condensates and induce their endocytosis. Importantly, this augmented general endocytosis is indispensable for stress responses such as reactive oxygen species (ROS) production and plant growth recovery after salt stress. The mechanism of salt induction of RALF–pectin condensation is likely to involve the altered apoplastic microenvironment with elevated RALF and de-esterified pectin (Liu et al., 2024b). Stress-induced nanoclustering appears to be widespread for membrane-associated proteins, especially in response to environmental stimuli. However, whether the formation of stress-induced nanoclustering shares the same mechanism as that outlined in this study remains to be explored. Nevertheless, this study provides an elegant example of how condensation at the outer layer of the cell is used to respond to osmotic and salt stress.

Intracellular changes caused by osmotic stress can also be perceived by protein condensation. SEUSS (SEU), a transcriptional regulator in *Arabidopsis*, could rapidly form osmotic stress-induced condensates in the nucleus that depended on its N-terminal IDR1. IDR1 was proposed to act as a crowding sensor; it contains two predicted α-helices that resemble previously designed crowding-sensing modules (Boersma et al., 2015). The authors demonstrated that the IDR undergoes an open-to-closed conformational switch upon an increase in molecular crowding, which triggers SEU condensation during osmotic stress (Figure 4B) (Wang et al., 2022). An interesting observation was that, although SEU without IDR1 fully complemented the developmental defects of the *seu* knockout mutant, the same truncated version of the protein failed to rescue the stress-sensitive phenotype of the mutant, providing solid genetic evidence that proper condensation of SEU is crucial for osmotic stress tolerance. At the molecular level, loss of SEU compromised the expression of genes related to the osmotic stress response (Wang et al., 2022). Cytoplasmic DCP5 was also recently shown to undergo crowding-dependent condensation in *Arabidopsis* (Wang et al., 2024c). DCP5 harbors a long segment of IDR that exhibits molecular crowding-induced conformational change. DCP5 condensates could recruit and sequester a substantial number of mRNAs and proteins, thereby modulating the transcriptome and translatome to maintain the balance between normal growth and osmotic stress response. Notably, DCP5 condensates differ from the heat-induced SGs and are therefore termed DCP5-enriched osmotic SGs (DOSGs). The formation and disassembly of DOSGs occur rapidly, and this may be crucial for effective response and tolerance to osmotic stress (Figure 4C) (Wang et al., 2024c).

Crowding-dependent condensation has also been reported in mammalian cells. The with-no-lysine (WNK) kinases were shown to undergo phase separation in cells in a molecular crowding-dependent manner upon hypertonic stress. WNK condensates enable activation of a downstream signaling cascade, facilitating the recovery of cell volume (Boyd-Shiwarski et al., 2022). We envisage that other proteins may exist to sense changes in molecular crowding to a different extent and may undergo condensation, thereby eliciting downstream cellular responses.

Other condensates responsive to osmotic and/or salt stress are also crucial for stress tolerance. Shoot meristemless (STM) is a transcription factor vital for initiation and maintenance of the shoot apical meristem. Salt stress has been reported to enhance the condensation of STM, which correlates with stronger transcriptional activity of *STM*, leading to increased shoot branching for acclimation to salt stress (Figure 4E) (Cao et al., 2023). Salt stress delays the dissolution of photobodies via inhibition of FER kinase, affecting the abundance of phyB protein and thereby fine-tuning the balance between plant growth and stress tolerance (Figure 4F) (Liu et al., 2023c). Salt stress can induce damage to proteins, which are sorted into vacuoles for degradation via autophagosomes or multivesicular bodies (MVBs) to maintain cellular homeostasis (Marshall and Vierstra, 2018). A recent study showed that calcium-dependent lipid binding protein 1 interacts with ALG2-interacting protein X and forms salt-induced condensates on immature autophagosomes (Figure 4G). These condensates enhance recruitment of the endosomal sorting complex required for transport (ESCRT) machinery, which is important for the maturation of autophagosomes, and promote salt tolerance (Mosesso et al., 2024). The ESCRT machinery mediates endomembrane remodeling and biogenesis of intraluminal vesicles (ILVs) inside MVBs (Gao et al., 2017). FYVE domain protein required for endosomal sorting 1 (FREE1), a plant-specific component of the ESCRT machinery, was recently shown to form osmotic-responsive condensates. Using a combination of physical theory, mathematical modeling, cell biology, and plant genetics, the authors demonstrated that FREE1 condensates directly invaginate and scission membranes to produce ILVs via surface tension (Figure 4D). This process does not require ATP consumption or the participation of other ESCRT components (Figure 4D). Furthermore, functional FREE1 condensation is required for osmotic stress response (Wang et al., 2024b). This study provides new insights into the interplay between condensates and membranes, which can be widespread throughout cells. How stress induces the condensation of CaLB and FREE1 remains to be clarified.

It should be noted that, in addition to increasing osmotic stress, salt stress also increases ionic strength. Whether phase separation can sense ionic strength remains to be determined. This is not impossible, as electrostatic interactions, which play key roles in phase separation, can be affected by ionic strength, and biosensors of ionic strength in cells have been designed on this basis (Liu et al., 2017).

One direct consequence of hyperosmotic stress is the loss of water molecules from the cell. Because water acts as a solvent for biomolecules inside cells, a reduction in water could compromise membrane integrity, resulting in instability of cellular shape and structure, disrupting the three-dimensional structures of proteins, and impairing enzymatic activities (Zhao et al., 2020). However, little is known about how cells directly sense intracellular water status. Some clues came from a recent study showing that a seed-enriched protein, FLOE1, undergoes hydration-dependent phase separation (Dorone et al., 2021). During seed maturation and germination, the seed cells experience loss and gain of water, respectively, which elicit a cascade of biochemical and metabolic events (Sajeev et al., 2024). Before germination, a desiccated and quiescent state is crucial for avoiding unfavorable environmental conditions (Sano et al., 2016). Therefore, accurate sensing of water is essential for seed germination. FLOE1 is an uncharacterized protein found to form cytoplasmic condensates upon imbibition of seeds (Figure 4H) (Dorone et al., 2021). When seeds were imbibed under water-stressed conditions such as salt or osmotic stress, FLOE1 condensates dispersed, attenuating seed germination. Manipulating the material properties of FLOE1 condensates also affected seed germination. Therefore, hydration-dependent condensation of FLOE1 was proposed to serve as a water-potential sensor for seeds (Dorone et al., 2021). This study suggested that phase separation could act as a water-sensing mechanism. However, the molecular basis of water sensing has yet to be determined. It may be related to hydrophobic interactions that make key contributions to multivalency in phase separation. As also stated in this study, technical challenges remain in dealing with dry seeds, and experimental systems that mimic dry seeds need to be established in the future.

## Condensates associated with other environmental stimuli

ROS are widespread in cells. They are not only induced by stresses such as heat, cold, drought, aluminum toxicity, organic pollutants, and pathogens but also accumulate during normal growth and development. Because of their strong oxidizing properties, excessive ROS cause cell damage. Yet at a proper level, ROS can act as signaling molecules, triggering signal transduction pathways in response to stress (Huang et al., 2019). Biomolecular condensation provides a means of sensing and responding to ROS. In the peripheral region of the tomato shoot apical meristem, cysteine residues of the transcription factor terminating flower (TMF) are oxidized by ROS, leading to an increase in inter- and intramolecular disulfide bonds, which subsequently triggers TMF phase separation. TMF condensates sequester the floral meristem differentiation gene *ANATHA*, ensuring precise flowering time (Huang et al., 2021b). ROS may also regulate condensation by oxidizing the methionine residues within IDRs (Kato et al., 2019). Condensates may have a widespread role in ROS signaling under both normal growth and various stress conditions.

In agricultural production, nitrogen deficiency is a common stress that elicits programmed cell death and senescence (Havé et al., 2017). Under nitrogen starvation, the Mediator subunit 19A (MED19a) shows reduced lysine acetylation that regulates its phase separation. The C-terminal IDR of MED19 is crucial for phase separation and interaction with the transcription factor ORESARA1 (ORE1), forming transcriptional condensates that positively regulate the expression of ORE1 target genes, ultimately leading to leaf senescence (Cheng et al., 2022). Given that various abiotic stresses can induce condensate formation in plant cells, we believe that numerous additional condensates involved in abiotic stress responses remain to be identified.

## Condensates in biotic stress

During the long-term arms race against pathogens, plants have evolved two immunity pathways to combat biotic stress (Jones and Dangl, 2006). Upon pathogen invasion, pathogen-associated molecular patterns (PAMPs) are recognized by transmembrane pattern recognition receptors, subsequently initiating cellular signal transduction and activating PAMP-triggered immunity (PTI) to limit pathogen colonization. To escape this immune response, pathogens deploy effectors into plant cells; these effectors are recognized by nucleotide binding and leucine-rich repeat domains proteins (NLRs), thereby inducing effector-triggered immunity (ETI). ETI usually leads to programmed cell death at the infection site, known as the hypersensitive response, to restrict the spread of pathogens. Despite different initiation mechanisms, both PTI and ETI share similar downstream responses that include kinase cascades, calcium influx, ROS accumulation, and transcriptional reprogramming. SA, an essential plant defense hormone, plays a vital role in coordinating responses to biotic stress (Jones and Dangl, 2006; Zhou and Zhang, 2020; Yuan et al., 2021). Recently, condensates have emerged as a widespread mechanism in plant immune response and regulation (Wang and Gu, 2022).

**Figure 5:**
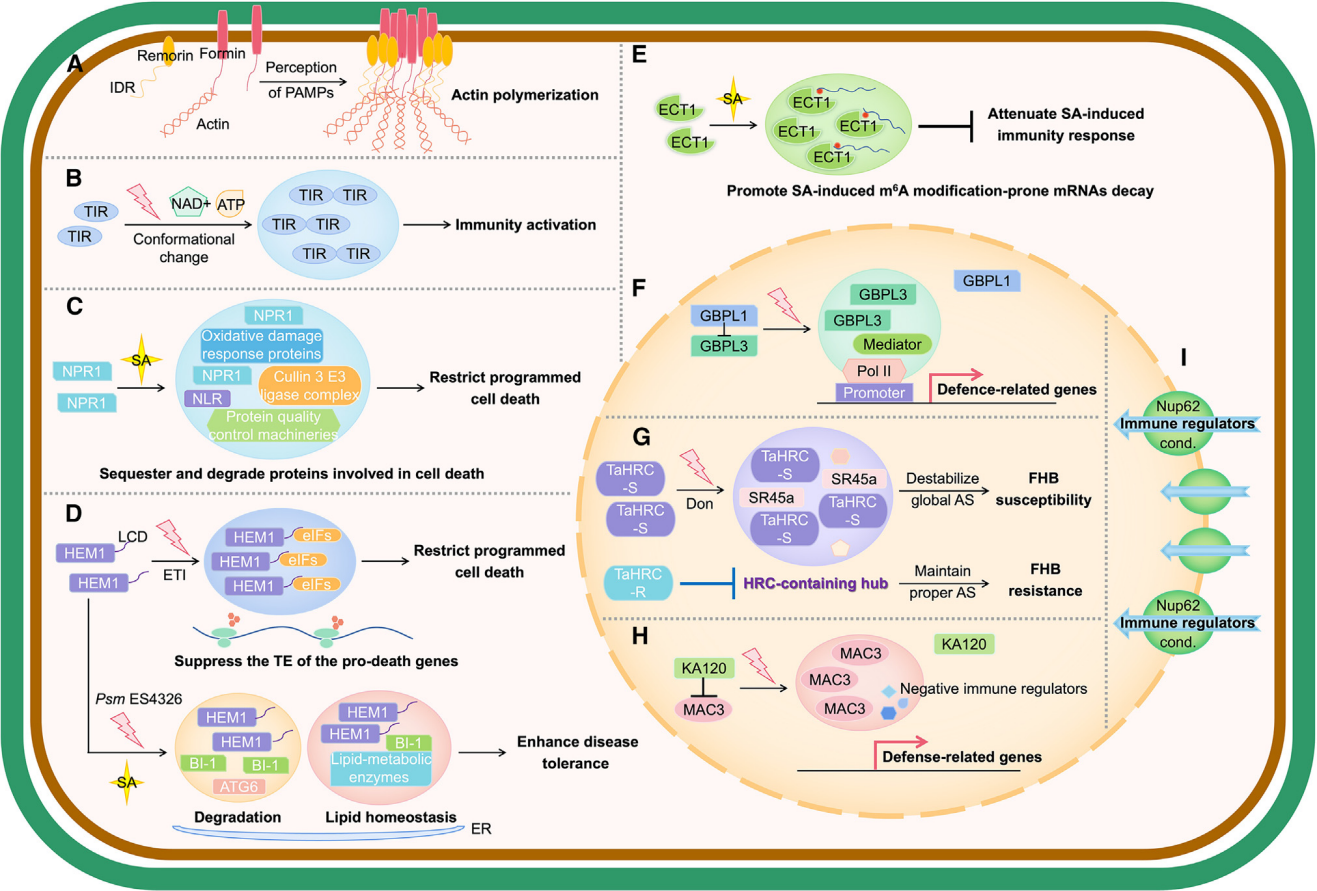
Condensates in plant biotic stress. **(A)** Upon receiving PAMP signals, formin nanoclusters undergo condensation mediated by remorin, subsequently leading to actin reorganization and fine-tuning of signal transduction. **(B)** TIR domain proteins undergo phase separation in a substrate-induced manner, which is essential for plant immunity. **(C)** NPR1 forms cytoplasmic condensates induced by SA; these condensates are enriched in stress response-related proteins and mediate the degradation of regulators that promote cell death, thereby enhancing plant survival under biotic stress. **(D)** HEM1 forms cytosolic condensates upon ETI activation; these condensates sequester translational machinery, thereby reducing the translation efficiency (TE) of pro-death immune genes. By contrast, HEM1 co-condenses with BI-1 at the endoplasmic reticulum during *Pseudomonas syringae* infection. BI-1 recruits autophagy-related proteins into the condensates, promoting their degradation. BI-1 also sequesters lipid-metabolism enzymes into the condensates, suggesting that lipid homeostasis may play a crucial role in plant disease tolerance. **(E)** Evolutionarily conserved C-terminal region 1 (ECT1) forms SA-induced cytoplasmic condensates that sequester SA-induced m^6^A modification-prone mRNAs and promote their decay, thereby attenuating the immune response to biotic stress. **(F)** The catalytic active regions of GBPL3 are sequestered by GBPL1 under normal conditions. Upon pathogen invasion, GBPL3 forms nuclear condensates that contain components of the Mediator complex and RNA polymerase II machinery, which directly bind to the promoters of disease resistance-related genes and enhance their transcription. **(G)** HRC-S forms condensates in the nucleus in response to the mycotoxin DON, which appear to contain splicing factors such as SR45a and modulate global alternative splicing, leading to an FHB-susceptible phenotype, whereas HRC-R does not. **(H)** The MAC complex undergoes condensation in the nucleoplasm upon pathogen infection, which can activate defense gene expression, presumably by sequestering negative immune regulators. KA120 suppresses MAC condensation under normal conditions. **(I)** Nup62 forms hydrogel structures that confer selectivity against immune regulators, which is essential for resistance to various biotic stresses.

During the PTI response, the actin cytoskeleton rapidly reorganizes. Upon receiving PAMP signals, formin proteins localize into nanoclusters on the membrane, a process that is positively correlated with actin assembly (Ma et al., 2021). Formin nanoclusters undergo condensation mediated by remorin, further increasing actin nucleation upon PTI activation (Figure 5A) (Ma et al., 2022). The nanoclustering and condensation of membrane proteins may represent a general mechanism for creation of spatially separated biochemical compartments that fine-tunes signal transduction during plant immunity.

Pathogenic effectors are recognized by intracellular NLR proteins. Effectors bind to the C-terminal domains of TIR–NLRs (TNLs) and induce the formation of tetrameric TNL resistosomes, activating their enzymatic activity (Chai et al., 2023). Monocot plants do not have these TNLs but instead contain TIR proteins that lack the C-terminal domain (Lapin et al., 2022). How effectors activate these TIR domain proteins is not clear. Recent research reported that TIR domain proteins undergo phase separation in a substrate-induced manner both *in vivo* and *in vitro* (Figure 5B). Mutational analyses showed that this condensation is positively correlated with the activation of TIR domain proteins. Using elegant structural data, the authors postulated that a substrate-binding-induced conformational change in a region called BB-loops mediates high-order TIR–TIR interactions and, subsequently, condensation. Genetic complementation experiments provided evidence that TIR condensation is essential for cell death and immunity (Song et al., 2024). This study provided a clear example of how condensates function in the activation of plant immunity .

As mentioned previously, ETI typically originates within infected local cells and promotes programmed cell death to limit the spread of pathogens. However, the activation of programmed cell death in neighboring cells with low pathogen loads is unnecessary and can adversely affect plant survival (Jones and Dangl, 2006; Bruggeman et al., 2015; Rodriguez et al., 2016). One mechanism to restrict excessive programmed cell death is SA-mediated systemic acquired resistance, in which nonexpressor of pathogenesis-related genes 1 (NPR1) plays a vital role (Fu and Dong, 2013). SA was found to induce the formation of cytoplasmic NPR1 condensates (SINCs) in plant cells (Figure 5C) (Zavaliev et al., 2020). Proteomic analyses revealed that SINCs are enriched in stress-response-related proteins such as NLR receptors and protein quality-control factors, as well as the E3 ligase complex. This suggests that, by mediating the degradation of cell death-promoting regulators, SINCs enhance plant survival under biotic stress (Zavaliev et al., 2020).

HEM1, also known as NCK-associated protein 1, is another factor that restricts programmed cell death during ETI. HEM1 forms cytosolic condensates upon ETI activation and sequesters translational machinery through its low-complexity domain, thereby reducing the translation efficiency of pro-death immune genes (Figure 5D) (Zhou et al., 2023). This mechanism helps plants to maintain a delicate balance between cell death to restrict pathogen spread and cell survival. A recent report showed that during *Pseudomonas syringae* infection, HEM1 interacts and co-condenses with BAX-inhibitor1 (BI-1) at the endoplasmic reticulum. BI-1 recruits autophagy-related proteins into condensates via protein–protein interaction, thereby promoting autophagic degradation of condensates (Figure 5D). Further studies found that lipid-metabolism enzymes could be sequestered into condensates through interactions with BI-1, potentially influencing lipid homeostasis, indicating that lipid homeostasis may play a vital role in plant disease tolerance (Tang et al., 2024). These two studies illustrate that the same condensate can have distinct functions depending on the client proteins as well as the subcellular location.

Although both SINCs and HEM1 condensates are localized in the cytoplasm and both contain translation-related factors, interaction of HEM1 and NPR1 was not observed in the condensates (Zhou et al., 2023), raising the intriguing question of how these condensates are temporally and spatially regulated.

*N*^6^-methyladenosine (m^6^A) has also been implicated in the SA-induced plant immunity response (Figure 5E) (Lee et al., 2024). Evolutionarily conserved C-terminal region 1, a key reader of m^6^A in *Arabidopsis*, forms SA-induced condensates in the cytoplasm through phase separation. These condensates sequestered SA-induced m^6^A modification-prone mRNAs and promoted their decay, subsequently attenuating the SA-induced immunity response to biotic stress (Lee et al., 2024).

Nuclear condensates are also reported to be important for immune responses. Guanylate-binding protein GTPases (GBPs) are known to enhance cell-autonomous immunity against pathogenic bacteria infection in mammals (Kim et al., 2011; Shenoy et al., 2012). A family of plant GBPLs that contain IDRs in their sequences were recently identified (Huang et al., 2021a). Under normal conditions, the catalytic active regions of GBPL3 were sequestered by GBPL1. Upon pathogen invasion, GBPL3 formed nuclear condensates termed GBPL defense-activated condensates (GDACs). GDACs contain components of the mediator complex and RNA polymerase II machinery, and GDAC formation is correlated with chromatin association. Notably, cryo-electron tomography of mammalian cells reconstituting GBPL3 condensates revealed that GDACs are likely surrounded by a chromatin boundary. Using genetic evidence, the authors postulated that GDACs directly bind to the promoters of genes related to disease resistance, subsequently promoting their transcription (Figure 5F) (Huang et al., 2021a).

The splicing regulatory protein complex MOS4-associated complex (MAC) was recently shown to undergo condensation in the nucleoplasm upon pathogen infection. These condensates can activate defense gene expression, presumably by sequestering negative immune regulators. Intriguingly, the authors found that KA120, a nuclear transport receptor that mediates the passage of macromolecules across the nuclear pore, suppresses MAC condensation under normal conditions (Figure 5H), likely controlling proper plant immune activation, although the detailed regulatory mechanisms underlying this process remain unclear (Jia et al., 2023). While this study suggests a noncanonical function of nuclear transport, the nuclear pore itself has been proposed to be a phase-separated compartment (Schmidt and Görlich, 2016). In plants, components of the nuclear pore complex (NPC) central barrier, Nup62/Nup58/Nup54, were reported to undergo phase separation, forming hydrogel structures that appeared to determine the permeability and selectivity of the NPC (Wang et al., 2023a). This study also showed that this NPC phase conferred selectivity against immune regulators such as MPK3 and that disruption of this phase property impaired resistance to various biotic stresses (Figure 5I) (Wang et al., 2023a).

In addition to their emerging role during bacterial infection, condensates have recently been implicated in the response to fungal infection in wheat. A recent study reported that the histidine-rich calcium-binding protein (HRC) condensates play a crucial role in resistance to FUSARIUM head blight (FHB), a devastating fungal disease of wheat that causes significant yield losses. HRC has two alleles, HRC-R (resistant) and HRC-S (susceptible), with HRC-R having an additional 14 amino acids at the N terminus (Li et al., 2019; Su et al., 2019; Hao et al., 2020). HRC-S formed condensates in the nucleus in response to the mycotoxin DON, leading to an FHB-susceptible phenotype, whereas HRC-R did not. HRC-S condensates appeared to contain splicing factors such as SR45a and to regulate the FHB response through the modulation of global alternative splicing (Figure 5G) (He et al., 2024). This study exemplifies how natural variation can affect phenotype via condensation.

## Future perspectives

Biomolecular condensates represent a new avenue for understanding stress perception and response in plants. However, what we know to date is only the tip of the iceberg. Below, we describe several potential research directions that can be explored in the future.

### Revealing the intracellular changes to which condensation can respond

As illustrated in Figure 1, numerous intracellular changes induced by extracellular stimuli might be perceived by phase separation but have not yet been reported. For example, changes in intracellular calcium and pH act as secondary messengers in response to different stress conditions, including salinity stress (Kader and Lindberg, 2010). A change in pH could affect protonation or deprotonation of certain proteins, influencing their intermolecular interactions and, subsequently, condensation. Yeast Sup35 has been reported to sense cellular pH under stress conditions and undergo liquid-like phase separation (Franzmann et al., 2018). The binding of calcium to calmodulin causes a conformational change from a closed to an open state, resulting in exposure of hydrophobic sites (Park et al., 2008) that could mediate multivalent interactions during condensation. Whether certain calcium-binding proteins undergo calcium-dependent condensation in plants is a promising topic. Calcium-induced condensates could have multiple functions, such as the partitioning of calcium-dependent kinases, calcium storage, and buffering. Metabolites are key constituents of cells and change upon external stimuli. Some have been found to regulate biomolecular condensation. For example, binding of NAD^+^ induces conformational changes in TIR proteins, leading to their condensation (Song et al., 2024), and ROS promote the condensation of TMF by regulating disulfide bond formation (Huang et al., 2021b). However, our understanding of the role and function of chemical molecules in protein condensation remains limited.

Another example is the change in cytoplasmic or nucleoplasmic viscosity under stress conditions. Although this is a fundamental cellular property, it remains largely unexplored in plant stress perception and response. A recent study showed that budding yeast modulates viscosity in response to temperature and energy availability, serving as a stress response and homeostatic mechanism, and perturbations to viscoadaptation affect the condensation of SGs (Persson et al., 2020). Therefore, identifying condensates that are responsive to additional intracellular changes will expand our understanding of plant stress signaling.

### Searching for additional stress-responsive condensates

It is difficult to discover novel stress-responsive condensates without comprehensive interrogation of the proteome. A method based on the small molecule b-isox has been used to enrich proteins that are prone to condensation (Liu et al., 2023). The application of this method to more contexts, such as specific tissues or plants stimulated by environmental stress, should lead to the identification of more condensation-prone proteins. The next step will be to use an appropriate system to screen for and identify stress-responsive condensates. Wang et al. used unicellular yeast cells for this purpose (Wang et al., 2022). Mammalian cells are also frequently used for condensation analysis of plant proteins (Huang et al., 2021a; Chen et al., 2022). These heterologous systems offer excellent tools for dissecting condensation but also raise the issue of artificial results in some cases. Nevertheless, it is crucial to return to the plant system after discovering stress-responsive condensation in other systems. Alternative approaches, such as biochemical fractionation and proximity-based labeling, are also expected to expand the repertoire of stress-responsive condensates in the future.

### Exploring other aspects of condensates in stress perception and response

IDRs often function as sensory elements in these “stress sensor” proteins. Under normal conditions, IDRs typically adopt a more flexible or disordered conformation. However, under stress conditions, IDRs may undergo conformational changes that lead to a more compact structure, which in turn triggers protein condensation, as observed in DCP5, SEU, and TWA1 (Wang et al., 2022, 2024c; Bohn et al., 2024). It should be noted, however, that the detailed biophysical mechanisms underlying stress-induced condensation of most stress sensor proteins remain poorly understood, making it challenging to identify the common features among these proteins. We anticipate that future studies, aided by interdisciplinary techniques, will provide further insights and help to address this issue.

Currently reported condensates respond to stress mainly through the process of LLPS, creating a dynamic condensed phase that is distinct from the diffused phase. These liquid-like condensates could function as sites of transient storage, selective compartmentalization, and concentration buffering, increase local concentrations, or create capillary forces, etc. (Figure 1). However, the liquid-to-gel, gel-to-solid, and more directly liquid-to-solid phase transitions should not be neglected in stress perception and response. Under some circumstances, the solid rather than the liquid phase is functional. For instance, the *oskar* RNP granules in the developing oocyte of *Drosophila* must be in a solid state to ensure embryonic development, as the solid phase can enrich specific proteins (Bose et al., 2022). A recent study demonstrated that the liquid-to-solid phase transition of SEC14-like, a plasma membrane-bound lipid-binding protein in *Arabidopsis*, contributes to root development by mediating the polarity of polar proteins such as the auxin efflux carrier PIN2 (Liu et al., 2023b). It is entirely possible that certain stresses induce intracellular changes that can stabilize intermolecular interactions, thereby triggering aging of condensates (Figure 1). In humans, disease mutations have been reported to trigger and accelerate the liquid-to-solid phase transitions underlying pathological aggregation (Patel et al., 2015).

### Understanding the role of condensates in long-term stress response

The advantage of biomolecular condensation in stress perception and response is its fast speed over a short time. However, in the natural environment, stress often persists for a long period of time. This raises the interesting question of whether and how condensates function in long-term stress responses. Intuitively, we believe that liquid-to-solid phase transition is likely to occur because the interactions that mediate the initial phase separation will inevitably become stable if the stress trigger persists. If this is true, what is the function of these condensates in the long-term stress response? What is the fate of these condensates? A recent study provides some hints for the latter question. During the course of heat SG assembly in plants, the 26S proteasome is incorporated into SGs and ensures their disassembly during the recovery from heat stress (Xie et al., 2024). Therefore, one possibility is that biomolecular condensates will eventually be cleared under long-term stress conditions. Future studies should solve this puzzle.

### Engineering stress-resistant crops using condensates

Most stress-responsive condensates have been characterized in the model plant *Arabidopsis*, with only a few studies in crops. This gap poses both a challenge and an opportunity for using knowledge to guide crop engineering and breeding. For example, natural variations in *Arabidopsis* ELF3 and TWA1 were found to correlate with the temperature threshold for condensation (Jung et al., 2020; Bohn et al., 2024). How this information can be transferred to crops is worthy of investigation. With advances in genome editing tools, the identification and introduction of desirable mutations to alter the material properties of endogenous condensates should help to improve stress tolerance and enable the development of optimized crop varieties. With an improved understanding of the mechanisms that govern stress perception and response by endogenous condensates, it will be possible to design artificial phase-separation modules in the near future to aid in ideal crop breeding, thus promoting agricultural development in a green, safe, and sustainable manner.

## Funding

This work was funded by grants from the National Key Research and Development Program of China (2024YFA1308100), 10.13039/501100002855Ministry of Science and Technology of China (2022YFA1303400) and the 10.13039/501100001809National Natural Science Foundation of China (32450060) to X.F.

## Acknowledgments

No conflict of interest is declared.

## Author contributions

J.P., Y.Y., and X.F. discussed the outline of the manuscript; J.P. and Y.Y. drafted the manuscript; J.P., Y.Y., and X.F. reviewed and edited the manuscript.
